# Analysis of the Mitogenomes of Two Helotid Species Provides New Insights into the Phylogenetic Relationship of the Basal Cucujoidea (Insecta: Coleoptera)

**DOI:** 10.3390/biology12010135

**Published:** 2023-01-14

**Authors:** Jing Liu, Yuhang Yang, Zihan Yan, Haishan Wang, Ming Bai, Chengmin Shi, Jing Li

**Affiliations:** 1 College of Plant Protection, Hebei Agricultural University, Baoding 071000, China; 2 Key Laboratory of Zoological Systematics and Evolution, Institute of Zoology, Chinese Academy of Sciences, Beijing 100101, China

**Keywords:** Helotidae, mt genome, Cucujoidea, genome structure, phylogenetic relationship

## Abstract

**Simple Summary:**

The family Helotidae represents a unique and primitive group of Cucujoidea, with important implications in understanding the phylogeny of beetles. To better understand the characteristics of the helotid mitochondrial (mt) genome and the evolution of Cucujoidea, we sequenced and compared the first recorded Helotidae mt genomes to reveal their characteristics and reconstruct the phylogenetic relationships of 13 basal families of this group. Phylogenetic analysis of the mt genomes indicated the positions of seven families within Cucujoidea but did not statistically support the presence of the Erotylid series and the Nitidulid series as distinct groups in this superfamily. In the phylogenetic results, Helotidae and Protocucujidae are sister groups. This study provides a new phylogenetic hypothesis regarding the basal relations of Cucujoidea.

**Abstract:**

Helotid beetles are commonly found in places where sap flows from tree trunks and in crevices in bark. The Helotidae family is a rare and primitive group of Cucujoidea. To date, no complete mitochondrial (mt) genome has been sequenced for this family. To better understand the characteristics of the mt genome and the evolution of Cucujoidea, we sequenced and annotated the complete mt genomes of *Helota thoracica* (Ritsema, 1895) and *Helota yehi* Lee, 2017 using next-generation sequencing. These are the first record of Helotidae mt genomes. The RNA secondary structures of both species were also predicted in this study. The mt genomes of *H. thoracica* and *H. yehi* are circular, with total lengths of 16,112 bp and 16,401 bp, respectively. After comparing the mt genomes of *H. thoracica* and *H. yehi*, we observed the gene arrangement, codon usage patterns, base content, and RNA secondary structures of both species to be similar, which has also been noted in other Coleoptera insects. The nucleotide sequence of the coding regions and the control region has small differences. The phylogenetic analysis indicated that Helotidae and Protocucujidae are sister groups and revealed the relationship between seven families; however, the validity of the two series (Erotylid series and Nitidulid series) as larger groups in the superfamily was not supported. The mt phylogenomic relationships have strong statistical support. Therefore, the division of Cucujoidea into series should be re-examined. Our results will provide a better understanding of the mt genome and phylogeny of Helotidae and Cucujoidea and will provide valuable molecular markers for further genetic studies.

## 1. Introduction 

As a small family in the superfamily Cucujoidea, the Helotidae is distributed in the Palearctic, Oriental, and Afrotropical regions [1,2]. The earliest fossil record of this family was found in the Early Cretaceous [3] and was regarded as one of the earliest diverging members of Cucujoidea [4]. Members of Helotidae can be distinguished from other families by the wide pronotum base, distinctly convex posterior angles, and the yellow spot on each elytron [5]. Most species are brightly colored. The adults and larvae inhabit trees and have complex and diverse diets (various fruits and tree sap) [6].

The first studies on helotid beetles date to 1825, with the description of the genus *Helota*, which was placed into the Engidae family [7]. The family Helotidae was established by Reitter in 1876 [8]. Kirejtshuk (2000) divided the family into five genera, forming the latest classification system [3,9,10,11]. Then, 18 new species were described, and 43 species were confirmed as synonymous by Lee. There are currently 79 species [4,12,13,14,15].

Based on the classification studies, the phylogeny of this family has also been discussed. The similarities of the aedeagus and the labrum-epipharynx between the Helotidae and the Nitidulidae show a close relationship these families [9,16]. Morphological data (by Leschen et al.) showed that Helotidae has a close relationship with Protocucujidae, Monotomidae, and Erotylidae [17]. Moreover, molecular data of a few gene markers also supported the same conclusion [18,19].

When the Erotylid series and Nitidulid series were established by Robertson et al. [20], Helotidae was included in the Erotylid series. The Erotylid series included Helotidae, Protocucujidae, Sphindidae, and Erotylidae, and the Nitidulid series included Nitidulidae, Kateretidae, and Smicripidae. The divisions of the two series were also supported by McKenna, 2015 [21]. However, this result was not supported in later phylogenetic analyses by Zhang et al., 2018 and McKenna et al., 2019 [22,23]. In both analyses, the Erotylidae was found to be the earliest diverged lineage of the Erotylid+Nitidulid series. Nevertheless, the Helotidae was shown to be most closely related to the ‘Sphindidae-Protocucujidae’ clade. Although these families have been confirmed to be closely related groups in Cucujoidea, the phylogenetic relationships are unresolved.

Since the first molecular phylogenetic study of Helotidae, that was based on three molecular markers (*COI*, *16S*, and *18S* ribosomal DNA) [19], different numbers and types of molecular markers have been used to reconstruct the phylogeny of these families. For example, there have been many reconstructions of beetle relationships using more nuclear markers (e.g., 95 nuclear protein-coding genes in Zhang et al.’s study and 4818 genes from RNA sequencing (RNA-Seq), and genome skimming in McKenna et al.’s study) [22,23]. Moreover, the results are different when it comes to the relationships between these families.

Currently, the mitochondrial (mt) genome is widely used in species genetic and molecular evolution analysis because of its higher evolutionary rate and more conserved transcripts than most nuclear genes [24,25,26,27]. Compared with individual mt genes, the complete mt genome can provide more genetic information and some understanding of genome-level characteristics such as RNA secondary structure and gene arrangement of insect mt genome [28,29,30].

With the development of next-generation sequencing, a great number of complete mt genomes for species of Coleoptera have been sequenced. However, studies concerning Helotidae mt genomes remain scarce, and no Helotidae mt genome is available in GenBank (https://www.ncbi.nlm.nih.gov). Here, to better understand the characteristics of the Helotidae mt genome, we sequenced and annotated the mt genomes of *Helota thoracica* (Ritsema, 1895) and *Helota yehi* Lee, 2017 [31,32], which are the first recorded Helotidae mt genomes. The nucleotide composition, relative synonymous codon usage (RSCU), gene overlapand control regions of *H. thoracica* and *H. yehi* were analyzed. The tRNA and rRNA secondary structures were predicted and analyzed. The results lay the foundation for the genomic study of Helotidae and Cucujoidea. Furthermore, the phylogenetic relationships of these families were reconstructed.

## 2. Materials and Methods 

### 2.1. Sampling and Genomic DNA Extraction

One ♂ *H. thoracica* (adult) and one ♂ *H. yehi* (adult) were captured by Mr. Feng in Da yao Mountain (23.927446, 110.093801, Google maps), Jin xiu City, Guangxi Province, China, on 5 April 2021. The specimens were stored in absolute ethanol at −20 °C and deposited in the Biological Control Laboratory, College of Plant Protection, Hebei Agricultural University (HBAU and accession No. LB001 and No. LB002). The DNeasy Blood and Tissue kit (QIAGEN, Hilden, Germany) was used to extract the isolated genomic DNA. DNA quality and concentration were measured on the Nanodrop 2000 spectrophotometer.

### 2.2. Mitochondrial Genome Sequencing and Assembly

Sequencing libraries were generated using the TruSeq DNA Sample Preparation Kit (Illumina, San Diego, CA, USA) and the Template Prep Kit (Pacific Biosciences, Menlo Park, CA, USA) with an average insert size of 350 bp. The genome sequencing was then performed by the Personal Biotechnology Company (Shanghai, China) using the Pacific Bio sciences platform and the Illumina Miseq platform with 150 bp paired-end reads. The Illumina raw data output was checked individually in the software Fast QC v0.11.5 (http://www.bioinformatics.babraham.ac.uk/projects/fastqc (accessed on 30 June 2021) to obtain clean data, and then trimmed with Trimmomatic v0.32. [33,34].

The Linux system was used to complete the assembly of the mt genomes. The software GetOrganelle v1.7.7.0 and MitoZ v2.3 [35,36] was used to conduct the assembly based on the sequence of the related species. The Geneious v8.0.5 software and Mitos v2.0 [37,38] were used for annotation and then the results were checked by manual proofreading according the relative mt genomes.

### 2.3. Gene Analyses

The Geneious v8.0.5 software was used to align with homologous genes from *H. thoracica* and *H. yehi* [37]. The software MEGA v7.0 was used to calculate the A + T content, AT-skew, GC-skew, and relative synonymous codon usage (RSCU) for (PCG) analysis [39]. The bias of base usage was calculated by AT-skew and GC-skew. The calculation formulas were as follows: AT-skew = (A − T)/(A + T) and GC-skew = (G − C)/(G + C). Tandem repeats in the control regions (CRs) were detected using the Tandem Repeat Finder v4.09 [40]. Twenty-two tRNAs were identified using tRNA-ScanSE Search Server v1.21 and ARWEN v1.2.3 based on the secondary structures and then were manually proofread according to the codon and tRNA structure [41,42]. The secondary structures of *rrnS* and *rrnL* were predicted by RNA Structure (http://rna.urmc.rochester.edu/RNAstructureWeb/) (accessed on 9 September 2021) and then the Clustal_W v2.1 algorithm in MEGA v7.0 was used to align them and the other available Cucujoid mt genomes [43]. The nucleotide diversity (Pi) of 13 Helotidae PCGs was assessed using DnaSP v6.0 [44]. A sliding window of 150 bp in 5 bp steps was performed using the Spider package in R v3.4.4 [45,46]. The software MEGA v7.0 [39] was used to calculate the genetic distances, based on the Kimura-2-parameter model, between the two mt genomes. The ratios of non-synonymous substitutions (Ka)/synonymous substitutions (Ks) for each PCG were measured using KaKs_Calculator v2.0 [47,48].

### 2.4. Phylogenetic Analyses

The phylogenetic analyses of these families were conducted based on 29 mt genomes from GenBank (http://www.ncbi.nlm.nih.gov (accessed on 10 November 2022) including the two newly sequenced genomes. The ingroup taxa included 28 species from Cucujoidea, representing 13 families. The outgroup Meloidae has a close relationship with Cucujoidea [20] (Table 1).

13 protein-coding genes were used to construct the dataset. The DNA alignment was performed from the amino acid alignment of the PCGs using the software Clustal_X v1.8.0 [49]. We connected all alignment sequences using MEGA v7.0.

The phylogenetic trees were reconstructed using different datasets under homogeneous and heterogeneous models. The homogeneous trees were reconstructed with maximum likelihood (ML) using IQ-Tree v1.6.8 [50] based on the dataset of PCG12. The PCG12 dataset includes 7190 sites for the first and second sites of the codon of 13 PCGs. Model Finder was used to select the model [51] (Table S1). Branch supports were evaluated using the ultra-fast bootstrapping method with 1000 replicates [52]. In addition, the heterogeneous tree was reconstructed using PhyloBayes v3.2 based on the dataset of PCG with the CAT-GTR model. The PCG dataset includes 10,785 sites for 13 PCGs. Two Markov chain Monte Carlo (MCMC) chains were employed [53]. FigTree v1.4.3 [54] was used to view and illustrate the inferred phylogenetic trees.

The approximately unbiased (AU) test and Shimodaira–Hasegawa (SH) test were used to evaluate the alternative phylogeny hypotheses, and CONSEL v0.1j and RaxML v8.2.4 were used for phylogenetic hypothesis testing. The per site log-likelihood was calculated with RaxML-master 8.2.4 using -f G (g). The *p*-values for each alternative hypothesis were estimated using the AU test and SH test implemented in CONSEL v0.1j [55,56,57].

## 3. Results

### 3.1. Genomic Organization and Base Compositions

**Figure 1:**
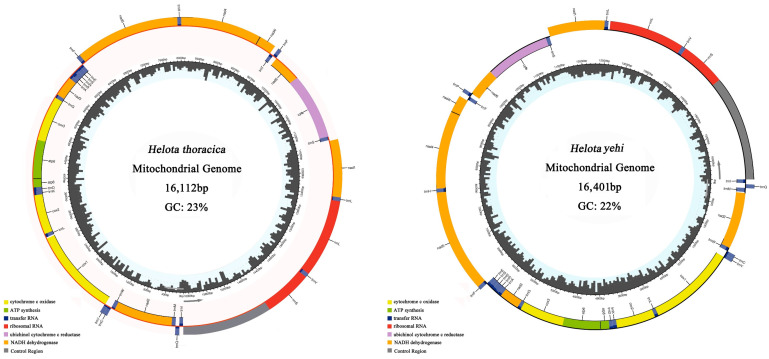
Mitochondrial map of *H. thoracica* and *H. yehi*. The cytochrome c oxidase (COX), ATP synthesis (ATP), transfer RNA (tRNAs), ribosomal RNA (rRNAs), ubichinol cytochrome c reductase (CYTB), NADH dehydrogenase (NADH) and control region (CR) are denoted by the different color blocks. The inside circles show the G + C contents of mitochondrial genome. Genes outside the map are transcribed counterclockwise, whereas those inside are transcribed clockwise.

The mt genomes of *H. thoracica* and *H. yehi* were typical double-stranded DNA molecules with sizes of 16,112 bp and 16,401 bp, respectively (Figure 1). They contained 37 genes, including 13 PCGs, 22 tRNA genes, 2 rRNA genes, and a control region (CR), all of which were consistent with other Cucujoidea mt genomes [58]. More genes were encoded on the majority strand (J), including 9 PCGs (*ATP6*, *ATP8*, *COX1*, *COX2*, *COX3*, *CYTB*, *ND2*, *ND3*, *ND6*), and 14 tRNAs (*tRNA^Ile^*, *tRNA^Met^*, *tRNA^Trp^*, *tRNA^Leu(UUR)^*, *tRNA^Lys^*, *tRNA^Asp^*, *tRNA^Gly^*, *tRNA^Ala^*, *tRNA^Arg^*, *tRNA^Asn^*, *tRNA^Ser(AGN)^*, *tRNA^Glu^*, *tRNA^Thr^*, *tRNA^Ser(UCN)^*), with the rest of the genes being oriented on the minority strand (N).

In these two mt genomes, there were overlapping nucleotides and non-coding regions. The conserved overlapping regions were located between *ATP8*/*ATP6* (4/7 bp), *ATP6*/*COX3* (1 bp), and *ND4*/*ND4L* (7 bp). Except the CR, in the non-coding regions of the mt genomes of the two species, *H. thoracica* has 53 bp of non-coding bases, while *H. yehi* includes 108 bp of non-coding bases (Table S1).

The complete mt genome records for these two species are the first for Helotidae. The annotated sequences of the two mt genomes were registered in GenBank with accession numbers OP964453 (*H. thoracica*) and OP964454 (*H. yehi*).

### 3.2. Base Composition

*Helota thoracica* and *H. yehi* were highly consistent in the analysis of A+T content, AT-skew, and GC-skew. The base composition and strand bias of these two species are shown in Table 2. The Helotidae mt genomes exhibited a significant bias towards A and T, with nucleotide compositions of A = 39.0%, C = 14.9%, G = 9.9%, and T = 36.2% for *H. thoracica*, and A = 39.6%, C = 13.4%, G = 9.4%, and T = 37.6% for *H. yehi*.

The nucleotide compositions of A and T in total ranging were from 77.00% in *H. thoracica* to 77.91% in *H. yehi*. The AT-skew was 0.05/−0.03 and the GC-skew was −0.23/−0.20. However, the content of A+T was the lowest in the PCGs, ranging from 74.97% in *H. thoracica* and 75.84% in *H. yehi*. Similar to other Coleoptera mt genomes, the content of A+T was the highest in CR, far exceeding the other features. The A+T contents of rRNA were second, and, in the two rRNAs, the A+T content of *rrnL* was significantly higher than that of *rrnS*.

Similarly, the mt genomes of *H. thoracica* and *H. yehi* exhibited positive AT-skews in tRNAs, CR and negative AT-skews in PCGs, rRNAs, positive GC-skews in all RNAs, and negative GC-skews in PCGs and CR. The PCGs, rRNAs, tRNAs, and CR had different AT-skews and GC-skews.

### 3.3. Protein-Coding Genes

The size of the 13 PCGs of *H. thoracica* was 11,120 bp. All the PCGs could be translated into 3698 amino acid residues. For *H. yehi*, the total size was 11,102 bp, which include 3691 amino acid residues. Similar to the CG, the PCGs exhibited a lower A+T content (74.97–75.84%). The AT-skew and GC-skew were both negative for the PCGs, reflecting a bias towards nucleotides T and C, as compared to their counterparts.

The majority of the PCGs started with ATN, except *ND1* in *H. thoracica* and *H. yehi*, which started with TTG. All PCGs stopped with TAA/TAG or truncated termination codons with T/TA-tRNA.

**Figure 2:**
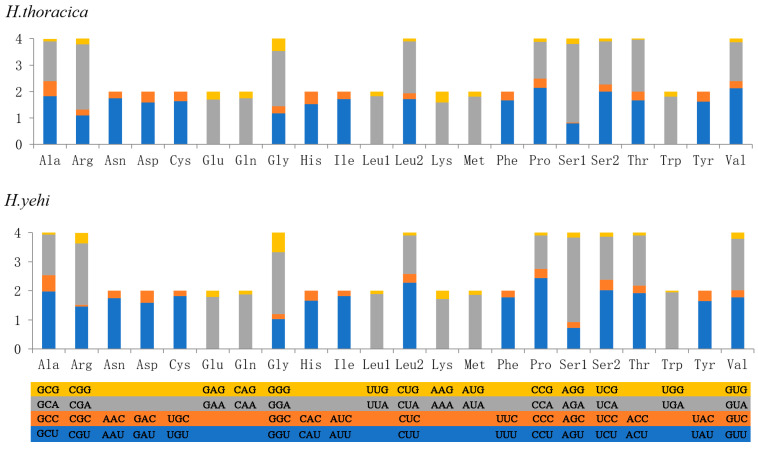
Relative synonymous codon usage (RSCU) of these 2 species. Codon families are provided on the *x*-axis.

As shown in Figure 2, the most frequently used aa were Leucine (Leu), Isoleucine (Ile), Phenylalanine (Phe) and Methionine (Met), and the four most frequently used codons were TTA, ATT, TTT, and ATA. The RSCU values of the PCGs revealed that the frequency of A and U in the third site of these two species was higher than the frequency of C and G, which indicated the preference for the nucleotide composition A/T. 

**Figure 3:**
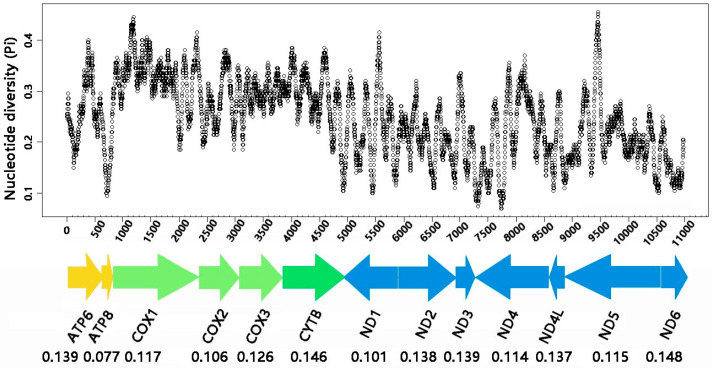
The nucleotide diversity (Pi) of 13 aligned PCGs of mt genomes among *H. thoracica* and *H. yehi* in a sliding window analysis (a sliding window of 150 bp with the step size of 5 bp). The line of small circles showed the value of Pi. Each gene names and the Pi values were shown in the graph.

The nucleotide diversity (Pi) of the 13 PCGs in *H. thoracica* and *H. yehi* was implemented using sliding window analysis (Figure 3). The Pi value ranges from 0.077 (*ATP8*) to 0.146 (*ND6*). *ND6* (Pi = 0.148) and *CYTB* (Pi = 0.146) exhibited significantly higher variability than the other PCGs, whereas the variability of *ATP8* (Pi = 0.077), *ND1* (Pi = 0.101), and *COX2* (Pi = 0.106) was relatively low and conserved 13 PCGs. However, as a useful marker for species identification, *COX1* (Pi = 0.117) indicated temperate conservativeness. The results indicated that the nucleotide diversity was varied among the 13 PCGs.

**Figure 4:**
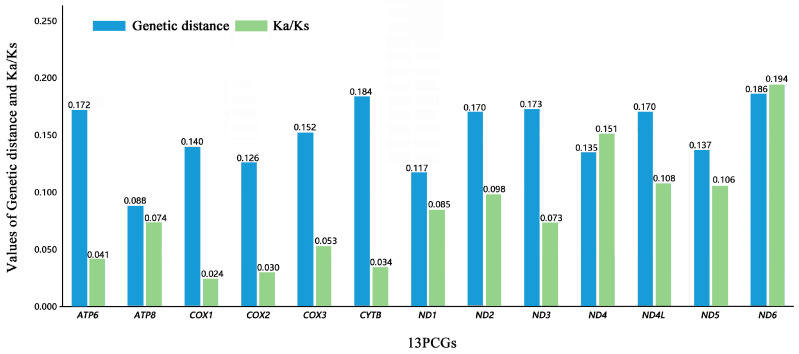
Genetic distance (on average) and non-synonymous (Ka) to synonymous (Ks) substitution rates of the 13 PCGs in *H. thoracica* and *H. yehi*. The average value for each PCG is shown in each histogram.

The analysis of pairwise genetic distance showed differing results, with *ND6* (0.186), *CYTB* (0.184), and *ND3* (0.173) having evolved relatively fast, and *ATP8* (0.088) and *ND1* (0.117) evolving slower (Figure 4). 

Similarly, the lowest genetic distance was not observed in *COX1*, with the genetic distance of *COX1* being 0.140. This result possibly indicates that *COX1* was not the most conservative gene in relation to PCGs in Helotidae. Average non-synonymous (Ka)/synonymous (Ks) ratios were estimated to investigate the evolutionary rates of mt genome PCGs [48]. We calculated the Ka/Ks ratios for each PCG of *H. thoracica* and *H. yehi* (Figure 4). The ratios ranged from 0.024 for *COX1* to 0.194 for *ND6,* in the following order: *COX1 < COX2 < CYTB < ATP6 < COX3 < ND3 < ATP8 < ND1 < ND2 < ND5 < ND4L < ND4 < ND6*. The average Ka/Ks of the 13 PCGs of these two species were all less than 1, which indicates that all the PCGs were under purifying selection. Purifying selection was particularly strong (Ka/Ks < 0.1) in the first nine coding regions of the order presented, with greater emphasis on the genes of complex III (*CYTB*) and IV (*COX1*, *COX2*, and *COX3*) in the mitogenomes. In particular, *COX1* (0.024) and *COX2* (0.030) were under the strongest purifying selection. The complex I genes (NADH) exhibited higher Ka/Ks proportions, especially in *ND6* (0.194) and *ND4* (0.151), which indicates the presence of less conservative evolutionary restrictions in these regions, which exhibited relaxed purifying selection. The results confirmed the pattern observed in previous studies, which also demonstrated heterogeneity among the evolutionary rates of different complexes encoding the mt genome.

### 3.4. Transfer RNAs

**Figure 5:**
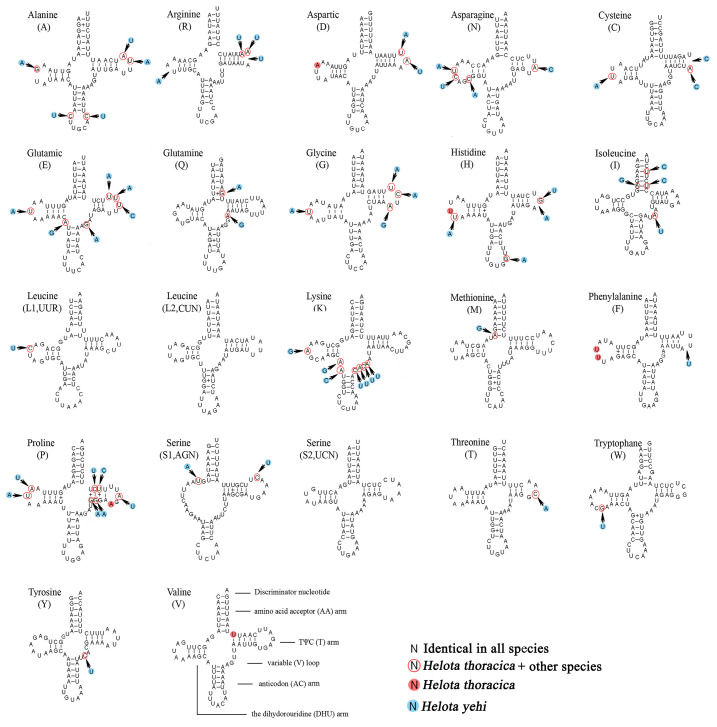
Inferred secondary structures of 22 tRNAs of *H. thoracica* and *H. yehi*. The tRNAs are labeled with the abbreviations of their corresponding amino acids. Dash (–) indicates Watson–Crick bonds and symbol (+) indicates GU bonds.

The secondary structures of tRNAs were predicted in *H. thoracica* and *H. yehi*, which are shown in Figure 5. The 22 tRNAs of these two species were both typical and included all 20 types of amino acids. Most tRNAs were highly consistent between *H. thoracica* and *H. yehi*. As a result of the two species being relatively similar and the tRNA genes being relatively conservative, the tRNAs of these two mt genomes were almost identical. The tRNA sizes ranged from 62 to 71 bp in *H. thoracica* and *H. yehi*.

Almost all of tRNAs could be folded into clover-leaf secondary structures, except *tRNA^Ser(AGN)^* whose DHU arm simply formed a loop. The anticodon of *tRNA^Ser (AGN)^* was UCU instead of GCU, which was used as the anticodon for metazoans.

In all predicted tRNA secondary structures, *H. thoracica* and *H. yehi* were highly consistent in terms of amino acid acceptor arm and loop, TψC arm and loop, anticodon (AC) arm and loop, and the dihydorouridine (DHU) arm and loop. Among them, these secondary structures of *tRNA^Leu(CUN)^* and *tRNA^Ser(UCN)^* were identical. The *tRNA^Tyr^*, *tRNA^Thr^*, *tRNA^Trp^*, and *tRNA^Met^* only exhibited a single base variation between *H. thoracica* and *H. yehi*. The aminoacyl (AA) stem length was 7 bp, which is conservative. The anticodon (AC) arm length was 5 bp, except for *tRNA^His^* and *tRNA^Leu(UUR)^*, and the AC arm was 4 bp. Almost all tRNAs had the same anticodon (AC) loop length (seven nucleotides), except for *tRNA^His^* and *tRNA^Leu(UUR)^* (nine nucleotides). The length of the TψC arm varied from 3 to 6 bp and the TψC loop from 3 to 8 nucleotides. The dihydrouridine (DHU) stem varied from 3 to 4 bp, except for *tRNA^Ser(AGN)^*, and DHU loop varied from 3 to 8 bp.

There are also base pair mismatches in both *H. thoracica* and *H. yehi*. Among them, the number of G-U mismatch pairs in the two species was the same, i.e., 15 G-U pairs, which form weak attraction and constitute bonds situated at the TψC arm (3 bp), the AA arm (3 bp), the AC arm (6 bp), and the DHU arm (3 bp).

### 3.5. Ribosomal RNAs

The *rrnL* was located in the *tRNA^Leu(CUN)^* and *tRNA^Val^*, and the length of *rrnL* ranged from 1258 (*H. yehi*) to 1286 bp (*H. thoracica*). The *rrnS* was located in the *tRNA^Val^* and the CR, and its length ranged from 759 (*H. yehi*) to 786 bp (*H. thoracica*). These rRNA (*rrnL*, *rrnS*) subunits were encoded on the N-strand.

The AT content ranged from 82.11% to 82.50% in *rrnL* and 79.30% to 79.97% in *rrnS*, which exhibited a high AT bias. The highest AT content in *rrnL* was found in *H. thoracica*, but the higher AT content in *rrnS* was found in *H. yehi*.

**Figure 6:**
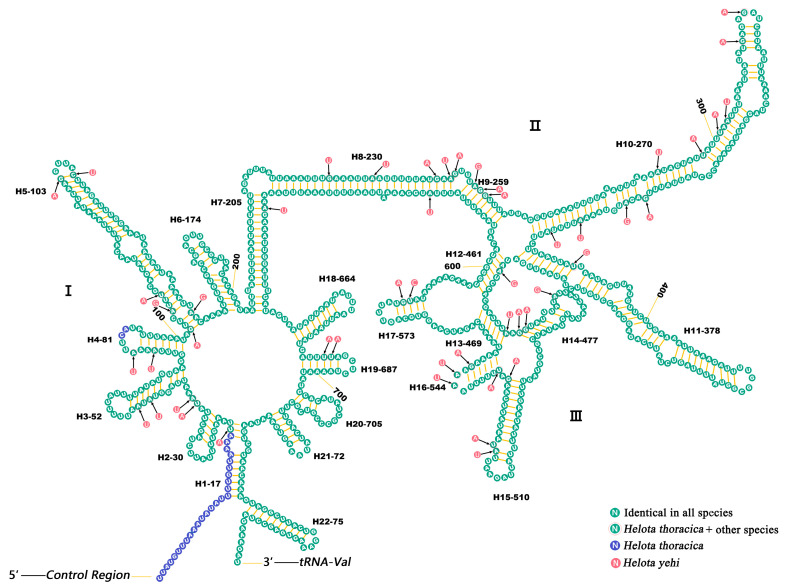
Predicted *rrnS* secondary structure in the mt genome of *H. thoracica* and *H. yehi*. Roman numerals refer to domain names. *Helota thoracica* is the base map and base changes in *H. yehi* are presented in circles with blue (*H. thoracica*) and gray (*H. yehi*) colors.

**Figure 7:**
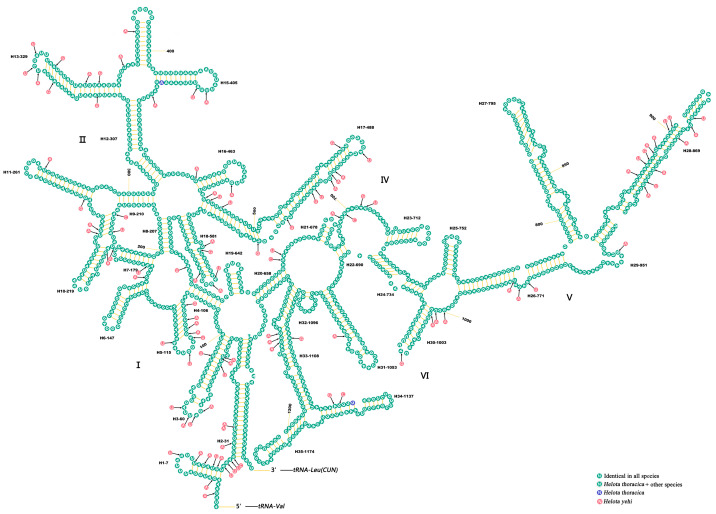
Predicted *rrnL* secondary structure in the mt genome of *H. thoracica* and *H. yehi*. Roman numerals refer to domain names. *H. thoracica* is the base map and base changes in *H. yehi* are presented in circles with blue (*H. thoracica*) and gray (*H. yehi*) colors.

The secondary structures of *rrnL* and *rrnS* were predicted and are shown in Figure 6 and Figure 7, respectively. The *rrnL* had 35 helices in five structural domains. The *rrnL* had five domains (I–II, IV–VI), except domain III, as is the case in Coleoptera insects [59].

The *rrnS* included three structural domains and 22 helices. However, the nucleotide conservation of two rRNAs was unevenly distributed among different domains. In *rrnL*, the domains IV and V were more conserved than in other domains, while the stem region of domain III was structurally more conserved in *rrnS*. 

### 3.6. Control Region

The control region plays an indispensable role in the analysis of molecular evolution, transcription, and contains regulatory functions for replication.

In Helotidae, the control regions were not conserved, but both were located between *rrnS* and *tRNA^Ile^*. The lengths of the CR in the two mt genomes were 1474 bp in *H. thoracica* and 1766 bp in *H. yehi*. The A+T content was 84.87% in *H. thoracica* and 85.73% in *H. yehi*. The A+T content of CRs was the highest, and both *H. thoracica* and *H. yehi* had positive AT-skews and negative GC-skews, which confirmed the characteristic in the Coleoptera mt genome.

**Figure 8:**
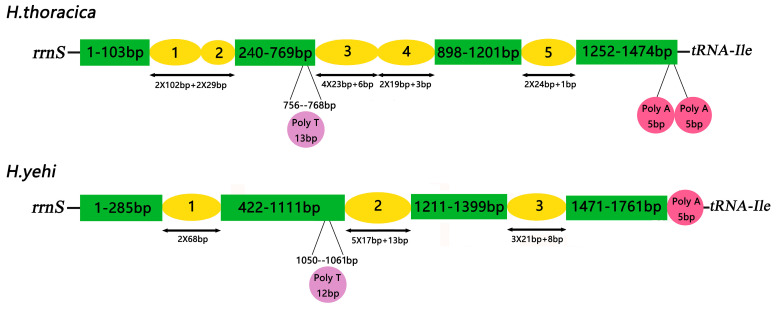
Organization of the control region in *Helota* mt genomes. The yellow ovals (number 1,2,3…) indicate the number of tandem repeats, the numbers below mean the sequence length (bp) of tandem repeats; the purple and red rounds indicate the Poly T/A; the non-repeat regions are shown with green boxes.

The Helotidae mt genomes had 3–5 types of tandem repeat units, ranging from 17 to 102 bp (Figure 8). Five tandem repeat units were found in the CR of the *H. thoracica* mt genome. They were a 29 bp, 19 bp, 24 bp, and 102 bp sequence tandemly repeated twice, and a 23 bp sequence tandemly repeated four times. In addition, the three tandem repeats in the *H. yehi* mt genome were a 68 bp tandemly repeated twice, a 17 bp tandemly repeated five times, and a 21 bp tandemly repeated three times.

**Figure 9:**
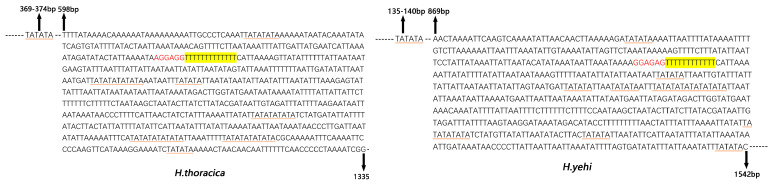
Features present in the control region of *H. thoracica* (**left**) and *H. yehi* (**right**).

There was conserved poly-A in the CR of both *H. thoracica* and *H. yehi,* upstream of *tRNA^Ile^*. The lengths of the poly-thymidine (Poly-T) structures were 13 bp in *H. thoracica* and 12 bp in *H. yehi*. The Poly-T stretch was an initiation of transcriptional control and replication. Moreover, there were many microsatellite-like repeat sequences, e.g., (TA) 6, (TA) 8, and (TA) 10, in the CR, and (TA) 10 only appeared in *H. thoracica* (Figure 9). Both the CRs included many short repeats, which may serve as microsatellites. These may be used to study the differences between individuals in different geographical locations and the phylogeny of Helotidae.

### 3.7. Phylogenetic Analyses

**Figure 10:**
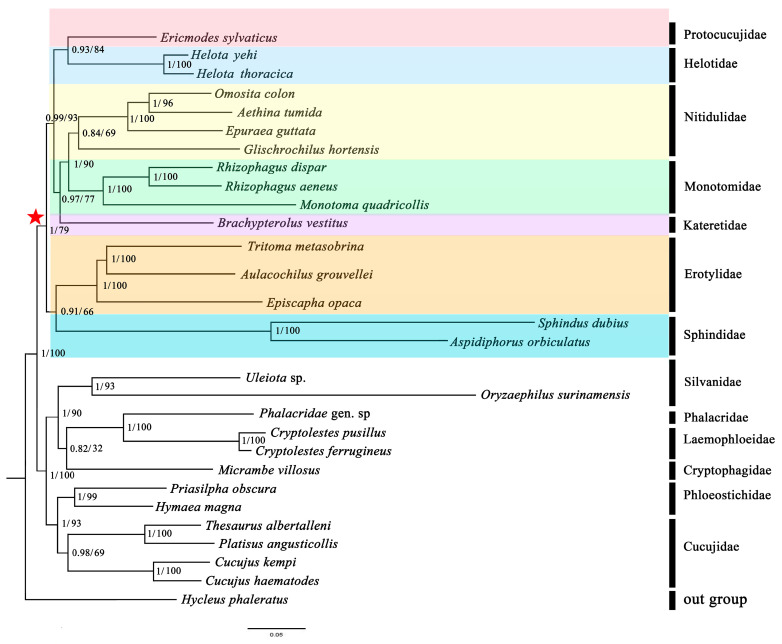
Phylogenetic relationships of the 13 basal families of Cucujoidea. Shown here is the phylogeny inferred from the PCG using Phylobayes v3.2 and the PCG12 dataset using IQ-tree v1.6.8. The number on the left represents the posterior probabilities (PP) from PhyloBayes v3.2 analysis and the number on the right represents ultrafast bootstrap support (BS, %) from IQ-Tree v1.6.8 analysis. The pentagram represents the location of phylogenetic relationships of the seven families.

Phylogenetic analyses were performed on the nucleotide datasets (PCG and PCG12). The phylogenetic results are shown in Figure 10. The analyses on the PCG dataset and the PCG12 dataset showed the same topology. Almost all of nodes were highly supported.

The Helotidae was defined as monophyletic and the sister group of Protocucujidae (Bayesian posterior probabilities, PP = 0.93 and ultrafast bootstrap support, BS = 84). Nitidulidae and Monotomidae were sister groups, which was together the sister group of Katertidae, and the PP and BS were mostly high. The sister group relationship between ((Nitidulidae-Monotomidae)-Katertidae) and (Helotidae-Protocucujidae) was highly supported in all analyses. The sister group, Erotylidae and Sphindidae, exhibited an obviously more distant relationship to the other groups. In this study, the monophyly of all these seven families was also supported.

**Figure 11:**
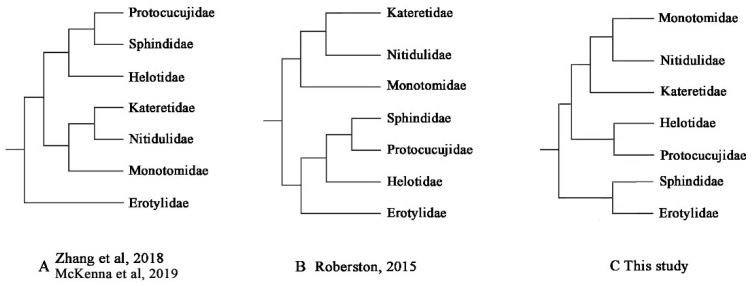
Family-level phylogeny hypothesis of seven families [22,23].

Therefore, after constructing the phylogenetic tree, the ML tree was used to statistically test the inconsistent phylogenetic hypotheses obtained by Zhang et al., 2018 and McKenna et al., 2019 (Hypothesis A), Robertson et al., 2015 (Hypothesis B) and this study (Hypothesis C) (Figure 11). The results are shown in Table 3.

The above results show that the *p*-values of the AU and SH tests were all less than 0.15 in other topologies, except in this study, indicating that there were significant differences between these studies. Under the mt genome dataset, only the result from our study was supported, which demonstrates that, at the mt genome level, the high-probability results are consistent with this research. Therefore, the results of this study show that the existence of two series is not supported at the mitogenome level.

## 4. Discussion

### 4.1. Comparative Analysis of the Two Helotid Mitogenomes 

Through Illumina DNA sequencing and assembly, the mt genomes of the two helotid species *H. thoracica* and *H. yehi* were obtained. With the exception of the diversity of the nucleotide composition, the mt genomes of these members of Helotidae were similar in terms of genome size, organization, arrangement patterns, gene order, aa compositions and RSCU to those of other Cucujoidea species [60]. The structural features were conserved. The majority of the PCGs started with ATN. The ND1 of these two species started with TTG. The TTG initiation has also been reported in other families such as Erotylidae and in other orders [58,61,62].

The analysis of evolutionary patterns showed that ND6 and CYTB exhibited a faster evolution rate, and ATP8 and ND1 exhibited the lowest genetic distance. As compared with non-synonymous substitution, the rates of synonymous substitution were significantly higher in all the PCGs (mainly in *COX1*) of the mitogenomes of the genus *Helota* analyzed herein. These were used as references for improved molecular mark development [63].

In addition, in the CR, these two mt genomes had a unique type of tandem repeat sequence units, but *H. thoracica* had two poly-As upstream of *tRNA^Ile^*, and *H. yehi* only had one. These features provide basic information for the further comparative analysis and discussion of Helotidae mt genomes.

### 4.2. Mitochondrial Phylogenomics Provides New Insights into Helotid Evolution

Although recent molecular phylogenetic studies have consistently recovered monophyletic suborders of Coleoptera and provided many new insights into the internal relationships of some suborders, the phylogenetic relationships within series and superfamilies of suborder Polyphaga still remain controversial [64]. This is particularly true for the superfamily Cucujoidea, within which the relationships among families were largely unresolved [20,21,22,23]. Our phylogenetic reconstructions at the mitochondrial level are consistent with the previous results of Zhang et al., 2018 [22], McKenna et al., 2019 [23], and Robertson et al., 2015 [20], that all showed that the seven families have a relatively close relationship. The results show that Helotidae forms a sister group to Protocucujidae, while Nitidulidae and Monotomidae are sister groups. Erotylidae and Sphindidae have a distant relationship to the other families. However, the sister groups among the families are in conflict with the results of previous studies. First, in our study, Protocucujidae and Helotidae are sister groups, the sister-group relationship between ‘Sphindidae-Protocucujidae’ and Helotidae is not supported. Second, although the Nitidulidae, Monotomidae, and Katertidae form a clade, as in previous studies, the sister group relationship is not the same. The research of Zhang et al., 2018 [22] and McKenna et al., 2019 [23] (hypothesis A), and Robertson et al., 2015 [20] (hypothesis B), supported ‘Nitidulidae-Katertidae’ and Monotomidae as sister groups, but our study suggests that Nitidulidae and Monotomidae are more closely related than Katertidae. Then, the phylogenetic results of mt genomes suggest that Erotylidae and Sphindidae have a close relationship, which also illustrates that our results do not support the existence of the Erotylid and Nitidulid series as divided by Robertson et al., 2015 (hypothesis B) [20]. This result has been statistically tested and does not support previous research based on nuclear protein-coding (NPC) and several molecular markers. This bias may originate from the genes in and the genetic differences between mt and nuclear genes [65,66]. However, one cannot easily reject the existing views based on information from one source of mt genome data. Multiple sources are necessary to make a final judgment. Therefore, we should take a balanced sample of the various taxa involved and conduct an in-depth discussion concerning this problem through the phylogenetic reconstruction of Cucujoidea within a larger sample range.

Compositional heterogeneity and evolution rate variability may be the most common sources of phylogenetic incongruence [67,68,69,70,71]. As a result of heterogeneity in the mt genome, dense taxon sampling and the model of CAT-GTR+G used, Bayesian analyses can produce robust phylogenetic trees though compositional heterogeneity cannot be eliminated [72]. Therefore, we used the CAT-GTR model in PhyloBayes to reconstruct the heterogeneous tree. In the maximum likelihood method, we select discarding/down weighting third codon positions to make the result more accurate [73,74].

## 5. Conclusions

In this study, two complete mt genomes of the family Helotidae were sequenced, they represent the first report of Helotidae mitochondrial genomes. This study opens a new phase in the study of genetic diversity among various families within Cucujoidea. At the same time, by reconstructing the phylogenetic relationships of 13 basal families in Cucujoidea, we propose a new view on the Erotylid and Nitidulid series. New insights into the phylogenetic position and evolution of the family Helotidae have also been provided. More data from other genera and species in the family are needed for further phylogenetics studies and to elucidate the molecular evolution of Cucujoidea. The mt genome sequences are important resources for further molecular studies and for the phylogenetic analysis of Helotidae and Cucujoidea.

## Figures and Tables

**Table 1:** List of taxonomic groups used for the phylogenetic analyses in this study.

Family	Species	Gene Bank Accession No.	Size (bp)
Ingroups			
Cryptophagidae	*Micrambe villosus* (Heer, 1841)	KX087317.1	17,907
Cucujidae	*Platisus angusticollis* Reitter, 1879	NC051936	15,921
Cucujidae	*Thesaurus albertalleni* Jin and Pang, 2020	MK614525	15,510
Cucujidae	*Cucujus kempi* Grouvelle, 1913	NC051939	15,492
Cucujidae	*Cucujus haematodes* Erichson, 1845	KX087268.1	16,120
Kateretidae	*Brachypterolus vestitus* (Kiesenwetter, 1850)	KX087245.1	16,536
Laemophloeidae	*Cryptolestes ferrugineus* (Stephens, 1831)	KT182067.1	15,511
Laemophloeidae	*Cryptolestes pusillus* (Schénherr, 1817)	NC028204.1	15,502
Monotomidae	*Rhizophagus dispar* (Paykull, 1800)	KX035133.1	13,423
Monotomidae	*Monotoma quadricollis* Aubé, 1837	NC036266.1	16,064
Monotomidae	*Rhizophagus aeneus* Richter, 1820	KX087340.1	16,454
Nitidulidae	*Epuraea guttata* (Olivier, 1811)	KX087289.1	16,021
Nitidulidae	*Aethina tumida* Murray, 1867	NC036104.1	16,576
Nitidulidae	*Omosita colon* (Linnaeus, 1758)	MW029385.1	16,544
Nitidulidae	*Glischrochilus hortensis* (Geoffroy, 1785)	JX412778.1	10,677
Silvanidae	*Uleiota* sp.	KX035149.1	14,967
Silvanidae	*Oryzaephilus surinamensis* (Linnaeus, 1758)	MN535903.1	15,941
Protocucujidae	*Ericmodes sylvaticus* (Philippi and Philippi, 1864)	KX035137.1	8404
Sphindidae	*Aspidiphorus orbiculatus* (Gyllenhal, 1808)	KT780625.1	18,500
Sphindidae	*Sphindus dubius* (Gyllenhal, 1808)	JX412803.1	10,509
Phloeostichidae	*Hymaea magna* Sen Gupta and Crowson, 1966	NC051933.1	16,888
Phloeostichidae	*Priasilpha obscura* Broun, 1893	EU877952.1	16,603
Phalacridae	Phalacridae gen. sp	MK614530.1	15,938
Erotylidae	*Aulacochilus grouvellei* Achard, 1923	MW291531	15,607
Erotylidae	*Tritoma metasobrina* Chûjȏ, 1941	MZ014622	16,502
Erotylidae	*Episcapha opaca* Heller, 1920	MZ014623	15,581
Helotidae	*Helota thoracica* (Ritsema, 1895)	OP964453	16,112
Helotidae	*Helota yehi* Lee, 2017	OP964454	16,401
Outgroup			
Meloidae	*Hycleus phaleratus* (Pallas, 1781)	MF491389.1	16,004

**Table 2:** Base composition and strand bias of these two species.

Feature	A + T (%)		AT Skew		GC Skew	
	*H. thoracica*	*H. yehi*	*H. thoracica*	*H. yehi*	*H. thoracica*	*H. yehi*
CG	77.00	77.91	0.05	−0.03	−0.23	0.20
PCGs	74.97	75.84	−0.13	−0.14	−0.04	−0.03
rRNA	81.32	81.31	−0.05	−0.04	0.34	0.31
*rrnL*	82.50	82.11	−0.06	−0.04	0.35	0.32
*rrnS*	79.30	79.97	−0.04	−0.05	0.32	0.29
tRNA	78.22	78.57	0.02	0.02	0.12	0.13
CR	84.87	85.73	0.01	0.02	−0.33	−0.33

Note: AT-skew = (A − T)/(A + T), GC-skew = (G − C)/(G + C); CG = complete mitogenome.

**Table 3:** Phylogenetic hypothesis testing.

Family-Level Phylogeny Hypothesis	AU	SH	KH	PP
A: Zhang et al., 2018 [22]; McKenna et al., 2019 [23]	0.123	0.127	0.127	4 × 10^−8^
B: Robertson et al., 2015 [20]	0.124	0.127	0.127	4 × 10^−8^
C: this study	0.877	0.873	0.873	1.000

Note: AU: Approximately unbiased test, SH: Shimodaira–Hasegawa test, KH: Kishino–Hasegawa test, PP: posterior probability.

## Data Availability

The data presented in this study are available in NCBI GenBank (Accession number: OP964453 and OP964454).

## Supplementary Materials

The following supporting information can be downloaded at https://www.mdpi.com/article/10.3390/biology12010135/s1: Table S1. Annotation of the mt genome of *Helota thoracica* and *Helota yehi.*
