# Supplementary material for: Analysis of the Mitogenomes of Two Helotid Species Provides New Insights into the Phylogenetic Relationship of the Basal Cucujoidea (Insecta: Coleoptera)
Source: Biology (Basel). 2023 Jan 14;12(1):135. doi: 10.3390/biology12010135 (PMC9855730; doi:10.3390/biology12010135)
Supplement: Supplementary file 1 [file biology-12-00135-s001.zip › biology-2129827-supplementary.pdf]

Table S1. Annotation of the mt genome of *Helota thoracica* and *Helota yehi.*

Gene	Direction	Location		Size	Anticodon	Codon		InteNgenic
		Start	End			Start	Stop	nucleotides
tRNA^{Ile}	J	1	64	64	GAU			-3/19
tRNA^{Gln}	N	62/84	130/152	69	UUG			-1/-2
tRNA^{Met}	J	130/151	198/221	69/71	CAU			0/17
ND2	J	199/239	1211/1234	1013/996		ATT/ATA	TA-tRNA/TAA	0
tRNA^{Trp}	J	1212/1235	1278/1301	67	UCA			0
tRNA^{Cys}	N	1279/1302	1341/1365	63/64	GCA			0
tRNA^{Tyr}	N	1342/1366	1406/1430	65	GUA			-8
COX1	J	1399/1423	2919/2970	1521/1548		ATT	T-tRNA/TAA	22/-6
tRNA$^{Leu(UUR)}$	J	2942/2965	3006/3031	65/67	UAA			0/-1
COX2	J	3007/3031	3694/3718	688		ATA/ATT	T-tRNA	0
tRNA^{Lys}	J	3695/3719	3765/3788	71/70	CUU			-1/0
tRNA^{Asp}	J	3765/3789	3830/3853	66/65	GUC			0
ATP8	J	3831/3854	3986/4009	156		ATT	TAG	-4/-7
ATP6	J	3983/4003	4654/4677	672/675		ATA/ATG	TAA	-1
COX3	J	4654/4677	5440/5463	787		ATG	T-tRNA	0
tRNA^{Gly}	J	5441/5464	5504/5527	64	UCC			0
ND3	J	5505/5528	5856/5879	352		ATT	T-tRNA	0

tRNA^{Ala}	J	5857/5880	5921/5944	65	UGC			0
tRNA^{Arg}	J	5922/5945	5984/6007	63	UCG			-1
tRNA^{Asn}	J	5984/6007	6047/6070	64	GUU			0
tRNA$^{Ser(AGN)}$	J	6048/6071	6114/6137	67	UCU			0
tRNA^{Glu}	J	6115/6138	6177/6200	63	UUC			0
tRNA^{Phe}	N	6178/6201	6243/6264	66/64	GAA			0
ND5	N	6244/6265	7961/7982	1718		ATT	TA-tRNA	0
tRNA^{His}	N	7962/7983	8026/8046	65/64	GUG			0
ND4	N	8027/8047	9357/9377	1331		ATG	TA-tRNA	-7/-7
ND4L	N	9351/9371	9635/9640	285/270		ATG/ATT	TAA	4/19
tRNA^{Thr}	J	9640/9660	9701/9721	62	UGU			0
tRNA^{Pro}	N	9702/9722	9766/9785	65/64	UGG			10/1
ND6	J	9777/9787	10262/10281	486/495		ATA	TAA	-1/5
CYTB	J	10262/10287	11399/11421	1138/1135		ATG/ATC	T-tRNA	0
tRNA$^{Ser(UCN)}$	J	11400/11422	11464/11486	65	UGA			17
ND1	N	11482/11504	12432/12454	951		TTG	TAG	0
tRNA$^{Leu(CUN)}$	N	12433/12455	12497/12519	65	UAG			0/30
rrnL	N	12498/12550	13783/13807	1286/1258				0
tRNA^{Val}	N	13784/1380	13852/13876	69	UAC			0

		8					
rrnS	N		13853/1387	14638/14635	786/759		0
		7					
Dloop			14639/1463	16112/16401	1474/1766		
		6					

Note: N and J indicate that the genes are located in the minor (N) and major (J) strand. The '/' indicated that these from left to right were *H.thoracica* and *H.yehi*. Intergenic sequence: positive numbers/negative numbers indicate intergenic/overlapping regions between adjacent genes.
